# Risk stratification in early non-ischaemic cardiomyopathy: the interplay of genetics and cardiac imaging

**DOI:** 10.3389/fcvm.2026.1890904

**Published:** 2026-10-06

**Authors:** Alexandros Kasiakogias, Christos Antoniou, Theodoros Tsampras, Freideriki-Eleni Kourti, Athanasios Kordalis, Alexios Antonopoulos, Ioannis Leontsinis, Charalambos Vlachopoulos, Constantina Aggeli, Konstantinos Tsioufis

**Affiliations:** First Department of Cardiology, Hippokration General Hospital, Medical School, National and Kapodistrian University of Athens, Athens, Greece

**Keywords:** artificial intelligence, cardiovascular magnetic resonance, dilated cardiomyopathy, genetic variant, late gadolinium enhancement, non-dilated cardiomyopathy, risk stratification, sudden cardiac death

## Abstract

Preventing sudden cardiac death (SCD) in early non-ischaemic cardiomyopathy (NICM) remains a major clinical challenge. Traditional risk stratification centred on left ventricular ejection fraction (LVEF) poorly captures arrhythmic risk, particularly in early dilated cardiomyopathy (DCM) and non-dilated left ventricular cardiomyopathy (NDLVC). Increasing recognition of fibrosis-based and genotype-specific arrhythmogenic substrates has prompted a shift towards multimodal risk assessment strategies integrating imaging, electrocardiographic, and genetic data. This review examines contemporary approaches to risk stratification in early NICM, with particular emphasis on the role of cardiovascular magnetic resonance (CMR) imaging and late gadolinium enhancement (LGE). The expanding role of genotype-specific phenotyping is also explored, including high-risk arrhythmogenic variants and the integration of genotype with imaging phenotype as a promising route to improved prediction. Collectively, these advances support a transition from population-based LVEF thresholds towards more personalised and biologically informed strategies for prevention of SCD in early NICM.

## Introduction

Non-ischaemic cardiomyopathy (NICM) encompasses a heterogeneous group of primary myocardial diseases that impose a substantial burden of sudden cardiac death (SCD), ventricular arrhythmias, and progressive heart failure (1, 2). Traditionally, risk stratification in this population has been anchored to left ventricular ejection fraction (LVEF), a paradigm shaped by landmark trials that predated contemporary cardiac imaging, systematic genetic testing, and refined phenotypic characterisation (3). Current clinical practice guidelines recommend an Implantable Cardioverter-Defibrillator (ICD) implantation for non-ischemic DCM with symptomatic heart failure and LVEF ≤ 35% after optimal guideline-directed medical therapy (GDMT). Yet, the LVEF is well-known to poorly capture arrhythmic risk. Reliance on an LVEF ≤ 35% captures only a subset of sudden cardiac death (SCD) cases, since most occur in patients with preserved or mildly reduced LVEF (4). The limitations of this LVEF-centric approach have been exposed most acutely in patients with early-NICM, namely those presenting with mild structural abnormalities that do not yet fulfil overt diagnostic criteria for dilated cardiomyopathy (DCM), in whom fibrotic and genetic substrates capable of driving life-threatening arrhythmias may already be operative despite preserved or only mildly impaired systolic function (5–7). This review discusses clinical definitions related to NICM and synthesises current evidence on risk stratification in early-NICM, examining the evolving role of clinical, imaging, and genetic markers (Figure 1). The aim is to guide a more individualised and comprehensive approach to identifying patients who may benefit from prophylactic intervention.

**Figure 1:**
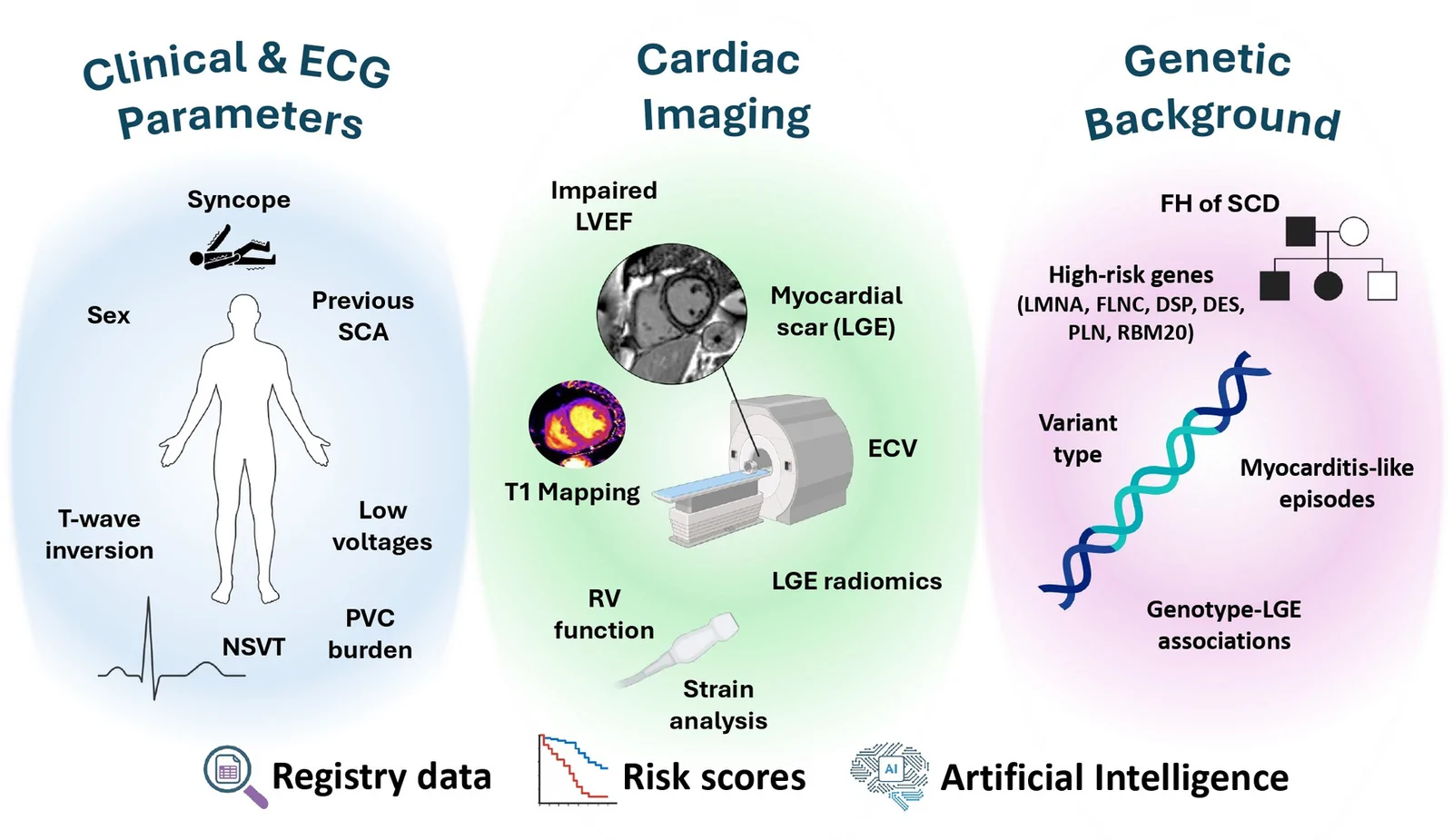
The three pillars of arrhythmic risk stratification in early non-ischaemic cardiomyopathy. Multiple parameters have been proposed to estimate the risk of life-threatening ventricular arrhythmias and sudden cardiac arrest, with varying levels of supporting evidence. These may be broadly categorized into: (i) clinical and electrocardiographic parameters, (ii) imaging-derived markers, and (iii) genetic and familial disease characteristics. The underlying genotype and gene-specific risk associations remain a major focus of ongoing research. Emerging approaches, including large-scale registry data, integrated clinical risk scores, and machine-learning algorithms, may further enhance individualized risk stratification and improve selection of patients for preventive therapies. PVC: premature ventricular contractions; ECG: electrocardiogram; NSVT: non-sustained ventricular tachycardia; SCA: sudden cardiac arrest; LVEF: left ventricular ejection fraction; RV: right ventricular; LGE: late gadolinium enhancement; ECV: extracellular volume; FH: family history; SCD: sudden cardiac death; LMNA: lamin A/C gene; FLNCtv: filamin C truncating variants; DSP: desmoplakin gene.

## Overview of current definitions

### Dilated cardiomyopathy and non-dilated left ventricular cardiomyopathy

Dilated cardiomyopathy is defined by left ventricular (LV) or biventricular dilation and systolic dysfunction that is not explained explicitly by abnormal loading conditions (e.g., hypertension, valvular disease, ventricular septal defect) or coronary artery disease (CAD). Presence of myocardial fibrosis is not included in the definition. A dilated LV is defined by end-diastolic diameters or volumes >2 z-scores over the population mean after adjusting for age, sex and body size. Typical transthoracic echocardiography thresholds for adults are an LV end- diastolic diameter >58 mm in males and >52 mm in females and a Left Ventricular End-Diastolic Volume (LVEDV) index of ≥75 mL/m^2^ in males and ≥62 mL/m^2^ in females (1, 8). CMR is the gold standard method for evaluation of ventricular size, and typical LVEDV index thresholds are ≥105 mL/m^2^ in males and ≥92 mL/m^2^ in females, but they should be based on available nomograms corrected by age, sex and body surface area (9). Left ventricular global systolic dysfunction is consensus driven and currently defined by LVEF <50% (in contrast to the previous cut-off of <45%) (1).

Non-dilated left ventricular cardiomyopathy (NDLVC) describes patients with normal LV size but presence of impaired global or regional systolic function with or without myocardial scarring and/or fatty infiltration (10). This phenotypic definition was formally introduced in the 2023 European Society of Cardiology (ESC) Guidelines for the Management of Cardiomyopathies, building upon the previously described term of Hypokinetic Non-Dilated LV (11). The reasoning behind this new terminology was the encounter of patients with cardiac imaging phenotypes that stand in-between a normal heart and DCM either as index cases or family members of patients diagnosed with a cardiomyopathy. Of note, the definition does not include the presence of ventricular arrhythmias or right ventricular (RV) involvement and at the same time requires CMR imaging for full characterization (12, 13). This phenotypic term should not be considered an end-diagnosis but should set the basis for further work-up. It is recognized that the NDLVC phenotype partially overlaps with the widely used term of arrhythmogenic LV cardiomyopathy (ALVC), itself proposed to define arrhythmogenic disease originating predominantly from the LV (14). The term scarring cardiomyopathy has also been proposed, to provide extra weight on the presence of myocardial fibrosis/scar as a substrate for potentially dangerous ventricular arrhythmias, irrespective of the underlying aetiology (15).

### Early non-ischaemic cardiomyopathy

Although the term NICM historically may have included all heart muscle diseases which are not a consequence of myocardial ischaemia or infarction, it is more widely applied to the non-ischaemic DCM phenotype (16). The term early non-ischaemic cardiomyopathy (early-NICM) is an umbrella term that describes mild abnormalities of cardiac structure and function not fulfilling the required features of the overt DCM definition. Perhaps currently more often used outside dedicated cardiomyopathy clinics, it is commonly used to describe individuals with mildly impaired LV function and/or mild LV dilatation with limited/absent fibrosis. The term describes patients that previously may not have been considered of significant arrhythmic or heart failure risk. However, it has been increasingly clear that they require detailed phenotyping and careful follow-up as a) there is a tangible risk of major adverse events despite the mild findings, and b) the disease progresses, sometimes at a fast rate (4).

The inception of the term early-NICM has several origins, and its etymological evolution reflects the rapid advancements in the field. The American College of Cardiology/American Heart Association Staging system introduced in 2001 describes Stage B or preclinical heart failure as the presence of structural heart disease and/or abnormal cardiac function or increased biomarkers without signs or symptoms of heart failure (17). A typical example is the patient with asymptomatic LV systolic dysfunction. In a seminal document published in 2016, preclinical or early phase DCM included patients who were genotype-positive/phenotype negative or had isolated LV dilation or presented with arrhythmias, albeit with no overt LV systolic dysfunction (11). A recent landmark investigation in early-NICM included three patient groups based on the presence and extent of hypokinesia and LV dilatation: The first subgroup comprised individuals with isolated LV dilatation, defined by increased LVEDV index in the presence of preserved systolic function (LVEF > 50%). The second subgroup consisted of patients with non-dilated LV cardiomyopathy, characterized by reduced LVEF despite normal LVEDV index. The third subgroup included patients with early DCM, defined by concomitant LV dilatation and mildly impaired systolic function, with LVEF values ≥50% but below the expected age- and sex-adjusted reference range (4). This definition excludes patients with improved/recovered LVEF or previous heart failure admissions.

## Risk stratification in non-ischaemic cardiomyopathy: the changing paradigm

For more than two decades, decisions regarding ICD implantation for primary prevention of SCD in patients with LV systolic dysfunction have been largely LVEF-centric. This paradigm was forged by landmark clinical trials conducted before advanced cardiac imaging, contemporary phenotyping and routine genetic testing were available (18–20). MADIT-II demonstrated a one-third reduction in all-cause mortality with ICD implantation, driven predominantly by SCD prevention, in post-myocardial infarction (MI) patients with LVEF ≤30%, irrespective of non-sustained ventricular tachycardia (NSVT) or inducibility on electrophysiology testing (19). Translation to the non-ischaemic substrate has proved problematic. SCD-HeFT enrolled symptomatic patients with LVEF ≤35% of equally split ischaemic and non-ischaemic aetiology and reported a 23% reduction in all-cause mortality with ICDs irrespective of cause; yet non-ischaemic disease was defined only by the absence of overt coronary artery disease, leaving the underlying substrate poorly characterised (21).

The DANISH trial questioned the use of LVEF alone for ICD selection in NICM, as among patients with non-ischaemic systolic heart failure and an LVEF ≤35%, ICD implantation did not result in a significant reduction in all-cause mortality (22). Extended follow-up has reinforced these findings documenting no overall mortality benefit but persistent SCD reduction, with the greatest benefit observed in sudden cardiovascular death in patients ≤70 years (HR 0.38; 95% CI 0.23–0.62) (23, 24). DEFINITE and CAT similarly failed to clearly demonstrate convincing survival gains, and meta-analyses have produced inconclusive results (25, 26). Collectively, these data establish LVEF as a necessary but insufficient discriminator of arrhythmic risk in NICM, particularly in early NICM, where overt LV dilatation and systolic impairment may not yet have developed but fibrotic and genetic substrates may already be operative.

In response, contemporary risk-stratification models embrace a multimodal “EF-plus” framework that variably integrates clinical, electrocardiographic, electrophysiological, imaging and genetic data (27, 28). Such an approach has been examined in the post-MI population: the PRESERVE-EF study showed that patients with LVEF >40% harbouring at least one non-invasive risk factor -NSVT, frequent premature ventricular contractions (PVCs), late potentials on signal-averaged electrocardiogram (ECG), prolonged QTc, abnormal T-wave alternans, reduced heart rate variability, or abnormal deceleration capacity/turbulence- had annual SCD rates of 0.6%–1%, with a two-step algorithm culminating in programmed ventricular stimulation achieving 100% sensitivity and 93.8% specificity for arrhythmic events (29). The ongoing RECONSIDER study is extending an analogous arrhythmic-substrate approach to non-ischaemic DCM (30).

The multimodal approach to risk stratification is supported by the fact that several clinical and electrical markers separately identify patients with NICM at risk. Unexplained syncope remains a plausible independent predictor of arrhythmic events (31, 32). Data from the Arrhythmia Cardiomyopathy Registry showed that Holter-detected NSVT and elevated PVC burden (i.e., ≥5-beat runs at ≥150 bpm, ≥1,000 PVCs/24 h or ≥50 couplets/24 h) define an arrhythmogenic phenotype that flags high-risk patients in NICM irrespective of LVEF. In a multicentre cohort of 742 DCM patients (median follow-up 6 years), this concept was refined with only NSVT and unexplained syncope -but not PVC or couplet burden- independently and additively predicting SCD/Major Ventricular Arrhythmias. Both markers together raised the 6-year arrhythmic event probability from 12% to 35% [incremental to LVEF and New York Heart Association (NYHA) class] and the prevalence of a high-risk arrhythmogenic genotype from 8% to 30% (31). NSVT appears to be most predictive when LVEF is greater than 35%, precisely the early-disease population in whom conventional ICD criteria do not apply (33). Resting ECG abnormalities (e.g., low QRS voltages, conduction delay, T-wave inversion) may provide further granularity (27, 34). Finally, in a prospective observational cohort of 254 patients with early NICM and a median LVEF of 55%, followed for a median of 7.9 years, independent predictors of the primary endpoint—including life-threatening ventricular arrhythmic events—were NSVT (HR 5.1), diabetes mellitus (HR 5.12), and myocardial fibrosis (HR 3.77) (4).

## Cardiac imaging for risk stratification: the role of late gadolinium enhancement

Late gadolinium enhancement (LGE) is present in roughly one-third of patients with NICM, most often as a mid-wall septal stripe, with subepicardial and ring-like patterns increasingly recognised in genetically determined disease (13, 35, 36). The 2023 ESC Cardiomyopathy Guidelines assign a Class I recommendation to CMR imaging at initial evaluation of patients with DCM and NDLVC, also reflecting the central role of LGE in diagnostic characterisation and risk stratification (1).

Across multiple observational studies and meta-analyses, LGE presence and extent in NICM is consistently associated with ventricular arrhythmias, sudden cardiac death, and adverse clinical outcomes (7, 37–40). In one of the largest meta-analyses to date (103 studies, 29,687 patients), LGE presence was independently associated with arrhythmic events (HR 2.69), all-cause mortality (HR 1.81), cardiovascular mortality (HR 2.43), and heart failure hospitalisation (HR 1.98) (37). LVEF was not independently associated with arrhythmic events or all-cause mortality. In the same study, quantitative LGE burden was additionally predictive (HR 1.07 per 1% LV mass increase). Indeed, the relationship appears continuous, with even minimal LGE conferring elevated risk and no clearly “safe” threshold. A landmark study showed a non-linear risk curve in which even very small LGE volumes (<2.55% of LV mass) were associated with significantly elevated SCD risk (HR 2.79). The risk curve showed the steepest inflection near zero LGE, implying that crossing from LGE-negative to any LGE presence represents a prognostically strong significant transition (41). These data are compelling but are almost entirely derived from retrospective, single-centre or registry cohorts with heterogeneous LGE acquisition, variable quantification methods and differing endpoint definitions, which limit pooled precision.

Beyond presence and burden, LGE distribution carries independent prognostic weight. A hierarchical risk gradient across LGE patterns has been observed across several cohorts. In a study of 874 DCM patients, septal-only LGE carried an approximately three times greater risk for SCD (41). Combined septal and free-wall LGE elevated this risk to nearly six-fold. At the same time subepicardial LGE (HR 5.54) and multiple LGE patterns (HR 5.72) presented with a significantly higher risk of SCD versus no LGE. All-cause mortality was increased with septal LGE (HR 2.00; 95% CI 1.43–2.81). These data support that additional or widespread LGE patterns confer substantially higher absolute and relative risk than isolated mid-wall septal fibrosis. In a multicentre, genotyped Spanish cohort of 577 patients, hazard ratios for major arrhythmic events versus no LGE ranged from 4.5 for transmural and 5.5–5.7 for linear mid-wall and subepicardial patterns to 18.2 for combined/multiple patterns. Importantly, LGE presence and extent were not correlated with LVEF in any genotype group (36). Such analyses are, however, constrained by inconsistent pattern definitions, limited reproducibility of qualitative scoring, and small subgroup event numbers.

The prognostic contribution of LGE is proportionally greatest in patients with preserved or mildly reduced LVEF- precisely the early-NICM population in whom conventional ICD criteria do not apply. In a retrospective cohort of 1,165 patients spanning the NICM spectrum (the median LVEF was 39%, interquartile range: 30%–46%), the LGE-positive versus LGE-negative hazard ratio rose to approximately 39-fold when LVEF exceeded 35%, versus about 3-fold at LVEF ≤20%, and an algorithm combining LGE, EF strata and high-risk distribution reclassified arrhythmic risk in 34% of patients beyond the LVEF ≤35% cut-off (42). In a pivotal study of 399 patients with mild DCM and LVEF ≥40%, LGE was associated with a higher risk of SCD (HR 4.9, 95% CI 1.8–13.5) and aborted SCD (HR 34.8, 95% CI 4.6–266.6), with 18-month event rates of 17.8% versus 1.1% in LGE-positive and LGE-negative patients, supporting CMR before excluding patients from ICD consideration based solely on LVEF (43, 44). Similarly, an observational study including derivation (*n* = 866) and validation (*n* = 848) cohorts of patients with NICM demonstrated that myocardial fibrosis outperformed LVEF for predicting SCD or ventricular arrhythmias (45). This study introduced a comprehensive fibrosis quantification framework distinguishing gray zone fibrosis (signal intensity 2–6 SD above remote myocardium from dense scar (>6 SD) and total fibrosis. Visual fibrosis independently predicted SCD and ventricular arrythmia events (HR 5.83, 95% CI 3.15–10.8), while total fibrosis >10 g identified patients at particularly high risk (HR 9.17, 95% CI 4.64–18.1). By comparison, LVEF <35% showed limited discriminatory value (HR 1.99, 95% CI 0.99–4.01). The recently published CMR GUIDE trial investigated whether LGE-guided primary-prevention ICD implantation improves outcomes in patients with mild-to-moderate LV systolic dysfunction (LVEF 36%–50%) and myocardial scar, compared with implantable loop recorder monitoring (46). The study did not demonstrate a significant reduction in the primary endpoint of SCD or haemodynamically significant ventricular arrhythmia with ICD implantation. Importantly, only 28% of patients had NICM, and eligibility in this subgroup was based on the presence of any LGE without consideration of scar extent or distribution, limiting conclusions regarding more refined LGE-based risk stratification in NICM. Further randomised evidence is needed to determine whether more refined LGE characterisation and its integration with other risk markers can effectively guide ICD selection and improve clinical outcomes in NICM.

## Towards a gene-specific approach to risk stratification

Genetic testing yields a causative or likely causative variant in 30%–40% of patients across the DCM/NDLVC spectrum, and genotype-positive status carries greater arrhythmic risk (6, 47–49). Causative variants in implicated genes also present with diverse arrhythmic risk profiles that are not closely associated with LVEF. Titin (TTN) truncating variants, the most common genetic cause of DCM (∼25% of familial cases), carry a comparatively lower arrhythmic risk and more favourable myocardial remodelling (48). Several studies have established that disease-causing variants in genes encoding for Lamin A/C (LMNA), Desmoplakin (DSP), Filamin C (FLNC), RNA-binding protein 20 (RBM20), Phospholamban (PLN), Desmin (DES) and others cluster into an arrhythmia-enriched phenotypic group, with markedly higher rates of SCD, sustained VT, and appropriate ICD therapy independent of LVEF (Figure 2) (47). This observation was formally recognised in the 2023 ESC Cardiomyopathy Guidelines and the 2022 ESC Guidelines on Ventricular Arrhythmias, which identify that disease-causing variants in genes encoding for LMNA, DSP, FLNC, RBM20, and PLN as high-risk genotypes warranting ICD evaluation beyond the LVEF ≤35% threshold (1, 50). The features summarised below show why a single risk framework cannot be applied uniformly across genotypes.

**Figure 2:**
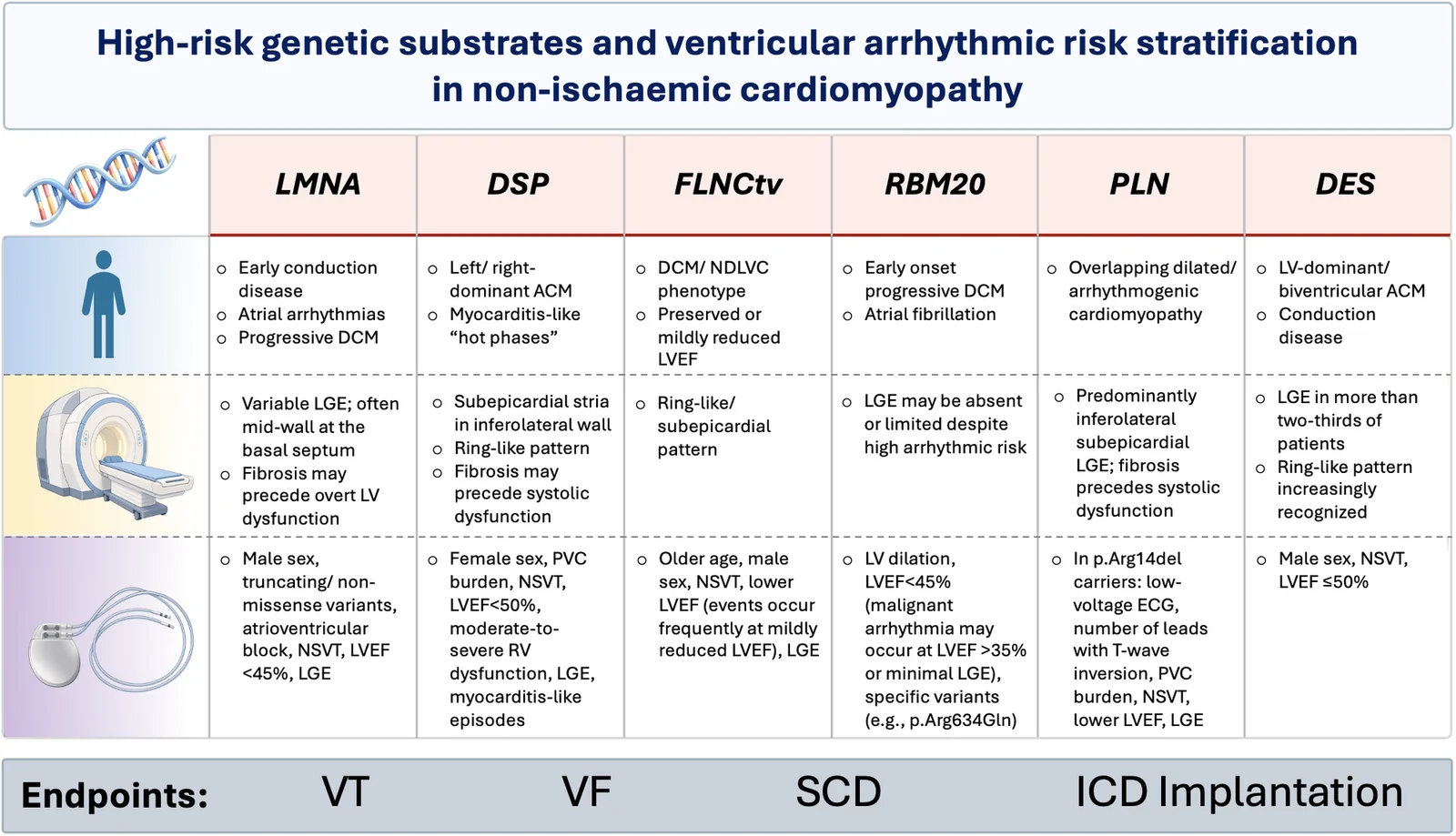
Schematic overview of major genes associated with increased ventricular arrhythmic risk in non-ischaemic cardiomyopathy. Typical phenotypic features, LGE findings and described risk markers of ventricular arrhythmias and sudden cardiac death are presented. Risk markers are based on available evidence from published cohorts and, where applicable, their incorporation into contemporary clinical practice guidelines. The list is not exhaustive, and some proposed risk markers require further validation. Notably, the magnitude of arrhythmic risk varies by gene involved and may be substantial despite preserved or only mildly impaired LVEF. Although LGE is regarded as a marker of arrhythmic risk, universally validated cut-offs for its extent and standardised criteria for high-risk LGE distribution across different genotypes remain lacking. Unexplained syncope is commonly considered a high-risk feature irrespective of genotype. LMNA, lamin A/C; DSP, desmoplakin; FLNCtv, filamin C truncating variant; RBM20, RNA-binding motif protein 20; PLN, phospholamban; DES, desmin; ACM, arrhythmogenic cardiomyopathy; DCM, dilated cardiomyopathy; NDLVC, non-dilated left ventricular cardiomyopathy; ECG, electrocardiogram; PVC, premature ventricular contraction; NSVT, non-sustained ventricular tachycardia; LV, left ventricular; RV, right ventricular; LVEF, left ventricular ejection fraction; LGE, late gadolinium enhancement; VT, ventricular tachycardia; VF, ventricular fibrillation; SCD, sudden cardiac death; ICD, implantable cardioverter-defibrillator.

LMNA causative variants, present in 5%–8% of DCM cases, destabilise nuclear envelope integrity and characteristically produce atrioventricular (AV) conduction disease or atrial arrhythmias before overt LV systolic dysfunction (51). Non-missense (truncating) variants carry a more malignant arrhythmic course than missense mutations. Van Rijsingen et al. identified four independent predictors of malignant ventricular arrhythmia: male sex, non-missense mutation, NSVT, and LVEF <45% (Table 1) (52). Wahbi et al. refined this observation into a validated five-variable model adding first-degree AV block and achieving a C-statistic of 0.80, with a ≥ 7% five-year arrhythmic risk threshold capturing 96.2% of life-threatening events (53). The CMR study of such patients frequently demonstrates mid-wall LGE involving the basal interventricular septum, a characteristic imaging phenotype associated with disease progression and adverse arrhythmic outcomes (54, 55). Supporting a role for fibrosis even before overt dysfunction, a prospective phantom-calibrated multicentre CMR study of 187 individuals found LGE in 28% of LMNA carriers with preserved LVEF (≥55%) versus none of the healthy volunteers. Across the entire cohort, over a median of 4 years, each 1% increase in global LGE extent was associated with a 15% higher risk of major adverse cardiovascular events (HR 1.15, 95% CI 1.02–1.30), and an LGE extent ≥7% conferred a 3.4-fold higher risk, whereas T1/T2 mapping added no incremental prognostic value. However, despite its recognized prognostic value, the presence, extent, or pattern of LGE has not yet been incorporated into validated LMNA-specific risk prediction models.

**Table 1:** Risk scores applicable in subpopulations of non-ischaemic cardiomyopathy.

Study	Gene/ model	Population (n)	Risk score parameters	Predicted risk/threshold	Endpoint
Peretto et al. (2026) (86)	Multigene NDLVC model	337 derivation; 216 validation	Male sex; NSVT; LVEF <45%; septal LGE; ring-like LGE; high-risk genotype; myocardial inflammation	5-year MAE risk categories: <5%, 5%–20%, 20%–40%, >40%	First major arrhythmic event: sustained VT, VF, or appropriate ICD therapy
Filamin C Registry Consortium (2025) (63)	FLNC truncating variants	308 carriers	Age; male sex; prior syncope; NSVT; LVEF	Individualized SCD/MVA risk prediction; no universally validated fixed threshold	SCD or major VA: aborted SCD, sustained VT, or appropriate ICD intervention
Carrick et al. (2024) (62)	DSP risk score	385 derivation; 86 validation	Female sex; NSVT; PVC burden; LVEF <50%; RV dysfunction	5-year VA risk categories: <5%, 5%–20%, >20%	Sustained VA (VT/VF), SCD, or appropriate ICD therapy
Verstraelen et al. (2021) (71)	PLN (p.Arg14del) model	679 carriers	LVEF; PVC count; number of leads with T-wave inversion; low-voltage ECG	5-year malignant VA risk: ≥5% (ICD consideration); ≥10%–15% (high risk)	Malignant VA: Sustained VT/VF, SCD, or appropriate ICD therapy
Wahbi et al. (2019) (53)	LMNA-risk VTA score	444 derivation; 145 validation	Male sex; non-missense LMNA variant; AV block; NSVT; LVEF	5-year life-threatening VTA risk ≥7% proposed for ICD consideration	Fatal/near-fatal VTA (SCD, appropriate ICD therapy, other manifestations of hemodynamically unstable VTA)

AV, atrioventricular; DSP, desmoplakin; FLNC, filamin C; ICD, implantable cardioverter-defibrillator; LGE, late gadolinium enhancement; LMNA, lamin A/C; LVEF, left ventricular ejection fraction; MAE, major arrhythmic events; NDLVC, non-dilated left ventricular cardiomyopathy; NSVT, non-sustained ventricular tachycardia; PVC, premature ventricular contraction; PV/LPV, pathogenic/likely pathogenic variant; RV, right ventricular; SCD, sudden cardiac death; VA, ventricular arrhythmia; VF, ventricular fibrillation; VT, ventricular tachycardia; VTA, ventricular tachyarrhythmia.

Clinical expression of variants in DSP and FLNC genes has been examined in detail in recent years. DSP pathogenic variants compromise desmosomal integrity, often driving a left-dominant cardiomyopathy phenotype with fibrosis, myocarditis-like “hot phases” of troponin-positive chest pain, and malignant ventricular arrhythmias (56, 57). Disease-causing variants of the Filamin C gene, a large actin-crosslinking cytoskeletal protein that stabilizes the sarcomere and links the contractile apparatus to membrane and mechanosignaling complexes, have also been shown to be associated with an elevated risk of arrhythmic events, and myocarditis-like episodes often despite preserved LVEF (58–61). A dedicated arrhythmic risk score based on data from the DESPERADOS registry incorporating female sex, NSVT, PVC burden, LVEF <50%, and RV dysfunction achieved a high negative predictive value for low-risk patient classification (62). In a cohort study of 308 individuals carrying FLNC pathogenic/likely pathogenic variants, those carrying truncating variants had a high arrhythmic risk and a 5-variable predictive model based on age, sex, syncope, NSVT, and LVEF improved the risk stratification of carriers of truncating FLNC variants (63). Both DSP and FLNC pathogenic variants are characteristically associated with subepicardial and ring-like LGE patterns (13, 36, 64). As with the LMNA risk score, LGE has not been incorporated into currently available gene-specific risk models for DSP or FLNC, although this should not be interpreted as evidence of limited prognostic significance. In the aforementioned DSP model, LGE was excluded because standardized quantitative assessment was unavailable across participating centres, CMR data were missing in approximately 20% of patients, and the predefined high-risk LGE pattern did not provide incremental prognostic value beyond the clinical variables included in the model (62).

Desmin is a muscle-specific intermediate filament protein that links the sarcomere to the cytoskeleton, nucleus, mitochondria, and intercalated disc. Pathogenic DES variants cause desmin aggregation and a pleiotropic cardiomyopathy phenotype overlapping with laminopathy-like conduction disease and DSP/FLNC-like arrhythmogenic left ventricular disease (65). In a large multicentre cohort of DES-related arrhythmogenic cardiomyopathy, Bermúdez-Jiménez et al. reported high penetrance, frequent LV-dominant or biventricular disease, substantial arrhythmic and heart-failure burden, LGE in more than two-thirds patients with a ring-like pattern in 49%, and adverse outcomes associated with male sex, NSVT, and LVEF ≤50% (66). The LGE pattern and clinical outcomes, particularly in male patients, closely resembled those observed in FLNC-related cardiomyopathy. Nevertheless, despite the high prevalence of ring-like LGE, adverse outcomes were associated with the aforementioned clinical parameters, rather than with the presence of LGE itself, which may reflect the high prevalence of LGE (approximately 70%) within the cohort, thereby limiting its discriminatory capacity (66).

RBM20 variants account for approximately 2%–6% of familial DCM and encode RNA-binding motif protein 20, a cardiac splicing factor that regulates titin and several calcium-/ion-handling genes, including CAMK2D and RYR2 (67, 68). RBM20 cardiomyopathy is characterised by early-onset DCM, frequent biventricular involvement, rapid heart-failure progression, and a disproportionately high ventricular arrhythmic burden compared with TTN-related DCM, often occurring before advanced LVEF reduction (68). Unlike desmosomal and cytoskeletal cardiomyopathies, RBM20 cardiomyopathy is characterized by little or no myocardial fibrosis, with LGE identified in only ∼5% of carriers placing it -together with TNNT2 and MYH7- in the “absent/rare” LGE category, without a characteristic subepicardial or ring-like signature (36). Yet arrhythmic risk is high, and driven by disturbed calcium handling rather than scar, so absent LGE should not be regarded as reassuring (36, 69).

Phospholamban is a key regulator of sarcoplasmic-reticulum calcium handling that modulates SERCA2a-mediated calcium reuptake during myocardial relaxation. The PLN p.Arg14del founder variant disrupts this regulatory pathway, leading to abnormal calcium cycling, myocardial fibrosis, proteotoxic stress, and a malignant overlap dilated/arrhythmogenic cardiomyopathy phenotype characterised by low QRS voltages, inferolateral T-wave inversion, ventricular arrhythmias often disproportionate to LVEF impairment, and progressive heart failure. Fibrosis is an early feature, present before systolic dysfunction and CMR frequently demonstrates inferolateral, predominantly subepicardial LV LGE, often with an accompanying linear mid-wall stripe in the septum (70). A PLN p.Arg14del-specific ventricular arrhythmia risk model has been developed incorporating LVEF, PVC burden, low-voltage ECG, and extent of negative T waves, which outperformed LVEF-based assessment alone; a subsequent analysis confirmed good model performance at 3-year reassessment, supporting repeated/dynamic ICD risk evaluation (71, 72). The presence and distribution of LGE have not been incorporated into the currently available PLN variant-specific risk model. Nevertheless, as myocardial fibrosis has been shown to be independently associated with ventricular arrhythmias in PLN cardiomyopathy, incorporating quantitative LGE assessment into future risk models appears a logical next step, pending further prospective validation (70).

## Discussion

Risk stratification in early NICM is evolving beyond reliance on isolated thresholds towards a multimodal framework integrating imaging, genetics, and computational modelling, although important uncertainties remain. Beyond the binary presence or absence of LGE and its quantitative burden, recent work has increasingly focused on the microarchitectural characteristics of myocardial fibrosis (40, 44). Scar heterogeneity is now recognised as a potentially important arrhythmogenic substrate in NICM. Radiomic analysis of LGE images, incorporating textural features such as entropy, contrast, and signal dispersion, has shown incremental predictive value over conventional scar quantification for ventricular arrhythmias (73, 74). Similarly, T1 mapping and extracellular volume quantification provide assessment of diffuse interstitial fibrosis, and recent meta-analytic data support their independent association with arrhythmic outcomes in NICM (75). Further evidence is needed to define the role of these emerging imaging markers in clinical practice and their incremental value for arrhythmic risk stratification beyond conventional LGE assessment (Figure 3, Table 2) (74, 76–82).

**Figure 3:**
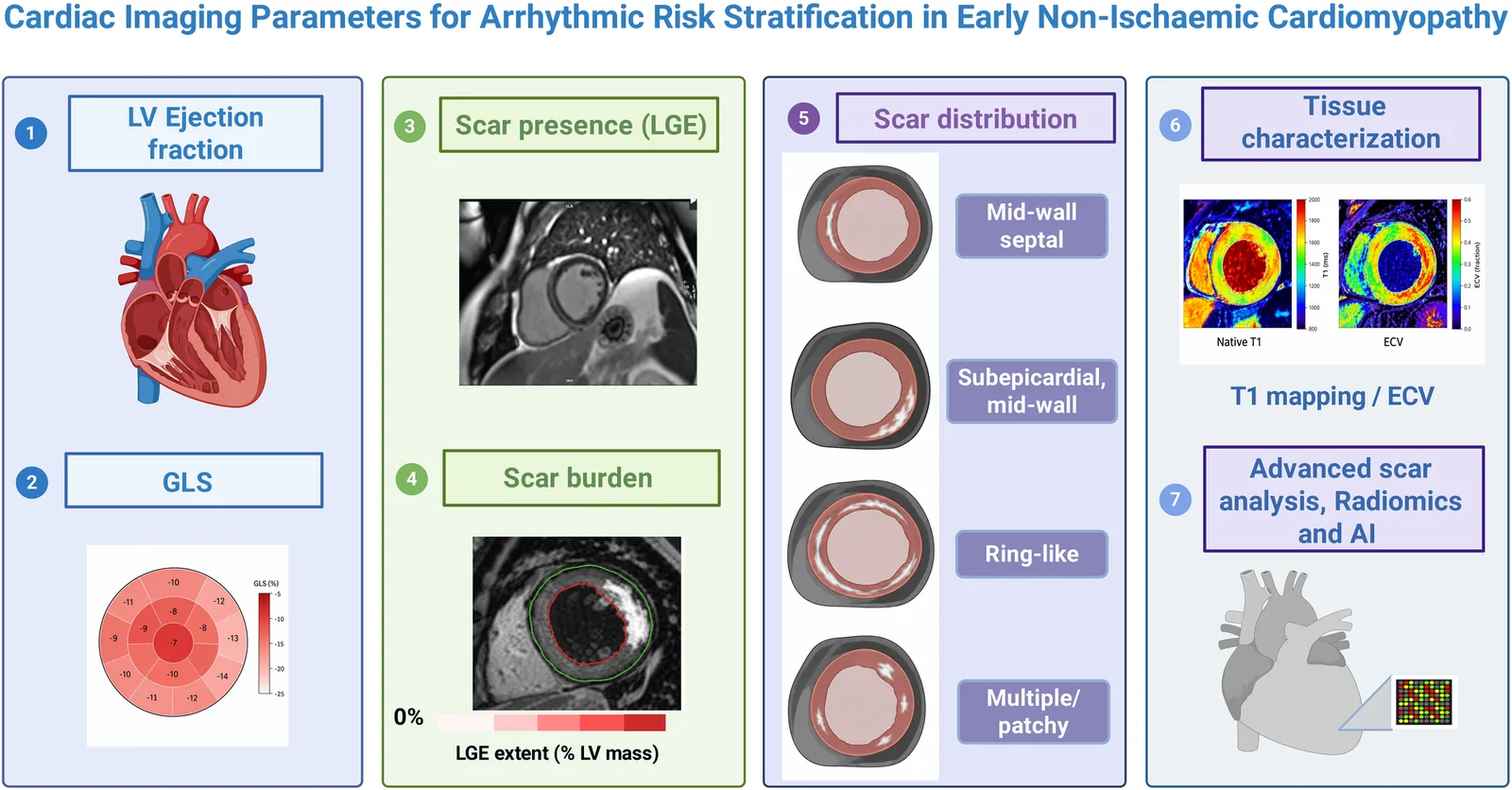
A focused overview of imaging parameters that may contribute to ventricular arrhythmic risk stratification in early non-ischaemic cardiomyopathy. These include left ventricular ejection fraction (LVEF), global longitudinal strain (GLS), and the presence, extent and distribution of late gadolinium enhancement (LGE). Tissue characterisation with native T1 mapping and extracellular volume (ECV), as well as advanced scar analysis using radiomic and artificial intelligence (AI)-based approaches, may provide additional information on the myocardial substrate, including scar texture and heterogeneity. The available evidence varies considerably across these parameters, and their optimal thresholds, incremental prognostic value and role in clinical risk stratification remain incompletely defined. Further validation is required before several of these approaches can be incorporated into routine risk assessment.

**Table 2:** Representative studies of potential CMR-derived imaging markers beyond conventional LGE for arrhythmic risk assessment in non-ischaemic dilated cardiomyopathy.

First author, year	Type of study	Population	Variable examined	Outcome	Main findings
Tissue characterization					
Cadour et al. (2023) (77)	Prospective multicentre cohort study	225 patients with NIDCM	Native T1, ECV, LGE	MACE, analysed separately as arrhythmia-related events (sudden death, sustained VT, appropriate ICD shock, resuscitated cardiac arrest, VF) and heart failure-related events	Native T1 Z-score >4.2 (HR 2.86, 95% CI 1.06–7.68) and ECV >30.5% (HR 2.72, 95% CI 1.01–7.36) were independent predictors of arrhythmia-related events.
Di Marco et al. (2023) (76)	Prospective single-center study (secondary analysis)	618 patients with NIDCM and available ECV	Native T1, ECV, LV-GLS	Composite arrhythmic endpoint: appropriate ICD therapy, sustained VT, resuscitated cardiac arrest, or sudden death	ECV ≥30% independently predicted ventricular arrhythmic events (HR 14.1, 95% CI 1.8–111.2).
Tissue heterogeneity and radiomics					
Hammersley et al. (2025) (74)	Prospective observational cohort study	291 patients with LGE-positive NICM	Fibrosis entropy (core, gray-zone and combined)	Life-threatening ventricular arrhythmias (SCD, aborted SCD, sustained VT)	Higher combined fibrosis entropy was independently associated with the endpoint, beyond LVEF and fibrosis burden (HR 1.98, 95% CI 1.30–3.02).
Nakamori et al. (2020) (78)	Prospective observational study	115 NICM patients evaluated for primary-prevention ICD	T1 mapping, T1 tissue Heterogeneity (Mad-SD)	Appropriate ICD therapy or SCD	Higher Mad-SD predicted arrhythmic events and provided risk stratification comparable to LGE, without requiring gadolinium.
Amyar et al. (2025) (79)	Two-centre retrospective cohort study with external validation	270 development and 113 validation patients with DCM and primary-prevention ICDs	LGE-derived radiomic features, including GLCM autocorrelation	Appropriate ICD therapy (shock or antitachycardia pacing) for VT/VF	Adding radiomic features to clinical variables and LGE improved discrimination. GLCM autocorrelation was independently predictive in both development and validation cohorts.
Myocardial deformation/mechanics					
Cojan-Minzat et al. (2020) (80)	Prospective cohort study	178 patients with newly diagnosed NIDCM	LV long-axis strain and LV sphericity index	MACE including heart failure events, VAs and SCD	Reduced LV long-axis strain and increased LV sphericity were independently associated with MACE. A 4-point score (LVEF <30%, LGE positivity, LAS >−7.8%, LVSI >0.48) stratified risk.
Ochs et al. (2021) (81)	Multicentre retrospective cohort study	350 patients with DCM	GLS, Global circumferential strain, Global radial strain, LV torsion	Composite endpoint of cardiac death, heart transplantation, or aborted SCD	GLS independently predicted the endpoint (HR 1.13, 95% CI 1.01–1.26).
Raafs et al. (2022) (82)	Observational cohort study	488 patients with DCM	LA reservoir and conduit strain, LV-GLS	Composite of sudden cardiac death, heart failure hospitalization, or life-threatening arrhythmias	LA conduit strain independently predicted adverse outcomes (HR 3.65, 95% CI 2.01–6.64) and provided incremental prognostic value beyond LGE and conventional functional parameters.

CMR, cardiovascular magnetic resonance; LGE, late gadolinium enhancement; NIDCM, non-ischaemic dilated cardiomyopathy; ECV, extracellular volume fraction; MACE, major adverse cardiovascular events; VT, ventricular tachycardia; ICD, implantable cardioverter-defibrillator; VF, ventricular fibrillation; HR, hazard ratio; CI, confidence interval; LV-GLS, left ventricular global longitudinal strain; NICM, non-ischaemic cardiomyopathy; SCD, sudden cardiac death; LVEF, left ventricular ejection fraction; Mad-SD, mean absolute deviation of the segmental pixel standard deviation from the average pixel standard deviation; DCM, dilated cardiomyopathy; GLCM, gray-level co-occurrence matrix; LV, left ventricular; VA, ventricular arrhythmia; LAS, long-axis strain; LVSI, left ventricular sphericity index; GLS, global longitudinal strain; LA, left atrial.

Genetic stratification has also advanced considerably, although substantial gaps remain. In particular, the within-gene granularity of risk is still incompletely defined. Distinguishing truncating from missense variants represents an important and still evolving area: truncating LMNA variants appear to confer a more malignant arrhythmic phenotype than missense variants, while variant location within DSP may influence risk in desmosomal cardiomyopathy (52, 83). Comparable variant-specific hierarchies for other disease-associated genes, however, still require further elucidation (67).

The emerging concept of “imagenetics”, integrating genotype with imaging phenotype, is especially compelling for arrhythmic risk prediction (84). Variants in DSP, FLNC, and dystrophin (DMD) are frequently associated with subepicardial and ring-like LGE patterns, reflecting disruption of desmosomal and cytoskeletal integrity (12, 34). In contrast, TTN truncating variants and BAG3-associated cardiomyopathy more commonly demonstrate non-specific mid-wall fibrosis patterns or limited detectable LGE, consistent with their comparatively less arrhythmogenic phenotype. One study identified ring-like subepicardial LGE in 78% of DSP/FLNC variant carriers, predominantly involving the basal and mid inferolateral LV walls, suggesting that this pattern may serve both as a diagnostic imaging signature for desmosomal/cytoskeletal cardiomyopathy and as a marker of increased arrhythmic risk (36). Nevertheless, several limitations temper the use of LGE for arrhythmic risk stratification in genetic cardiomyopathies for the time being. The available evidence comes from retrospective series at specialist referral centres, where individual genotype subgroups are small and arrhythmic events few, so risk estimates are easily overfitted and may not hold up on external validation. LGE acquisition and quantification remain non-standardized (e.g., visual versus signal-intensity thresholds) with limited reproducibility across vendors and populations. At the same time, CMR/LGE data are frequently incomplete or entirely absent in the cohorts used to derive gene-specific risk models, some of which omit imaging altogether, and confounders such as incomplete, age-dependent penetrance, endpoint heterogeneity, and competing risks (heart failure and non-cardiac death) are seldom accounted for (85).

Registry-based datasets are likely to play an important role in integrating these multidimensional data. Supporting this approach, a recently validated 5-year risk model for NDLVC incorporated male sex, LVEF <45%, NSVT, septal and ring-like LGE, high-risk genotype, and myocardial inflammation into a multiparametric score stratifying patients across four risk categories (<5% to >40% at five years) (86). Artificial intelligence is emerging as an additional tool capable of integrating imaging, electrophysiological, genetic, and clinical information into patient-specific predictions. Early multimodal approaches integrating imaging phenotype, genetic data, ECG features, and clinical variables into unified risk models have shown promising discriminatory performance, although prospective validation and clinical implementation remain limited (87, 88). Despite its promise, the application of artificial intelligence to risk stratification in early NICM remains limited by several important challenges. Event rates are low in early disease, increasing the risk of model overfitting, while the rarity and biological heterogeneity of individual genotypes restrict the development of robust gene-specific algorithms. Current models are largely based on cross-sectional imaging and rarely fully integrate longitudinal changes, genetic information, ambulatory rhythm data, or circulating biomarkers. In addition, limited external validation, variability in imaging acquisition, and the “black-box” nature of many deep-learning approaches continue to hinder clinical adoption (87).

In summary, risk assessment in early NICM is progressively transitioning from population-based metrics towards personalised, biologically informed models. Advances in fibrosis characterisation, complementary imaging biomarkers, genotype-specific phenotyping, and machine-learning methodologies may allow earlier identification of the relatively small subgroup of patients with substantial arrhythmic risk, while helping to avoid unnecessary interventions in individuals with a more favourable clinical trajectory (Table 3).

**Table 3:** Key take-home messages for arrhythmic risk stratification in early non-ischaemic cardiomyopathy.

Domain	Key message
Beyond LVEF	LVEF alone is insufficient for arrhythmic risk stratification in early non-ischaemic cardiomyopathy, supporting a shift towards an “EF-plus” approach integrating clinical, electrical, imaging, and genetic risk markers.
Clinical and electrical phenotype	NSVT and unexplained syncope are important markers of arrhythmic risk and, alone or in combination, may provide incremental prognostic information when LV systolic function is preserved or only mildly impaired.
CMR and myocardial fibrosis	LGE is a key imaging marker of arrhythmic risk, with its presence, extent, and distribution providing prognostic information beyond LVEF. Even limited LGE may confer increased risk, while no universal quantitative LGE threshold has been established.
Genotype-specific risk	Pathogenic variants in high-risk genes including LMNA, DSP, FLNC, RBM20, and PLN may confer substantial ventricular arrhythmic risk even in the absence of severe LV systolic dysfunction, supporting genotype-specific risk assessment. Available gene-specific risk models should inform, but not replace, clinical judgment.
Genotype-imaging interplay	Integration of genotype and imaging phenotype may further refine risk assessment, while characteristic LGE patterns may provide clues to the underlying genetic substrate. However, the incremental prognostic value of imaging within individual genotypes remains incompletely established.
Personalized and dynamic risk assessment	A multifactorial approach may enable more personalised arrhythmic-risk assessment and better inform ICD decision-making than isolated LVEF thresholds. However, prospective evidence across different substrates remains limited. Arrhythmic risk is dynamic and should be reassessed during follow-up.

CMR, cardiovascular magnetic resonance; DSP, desmoplakin; FLNC, filamin C; ICD, implantable cardioverter-defibrillator; LGE, late gadolinium enhancement; LMNA, lamin A/C; LV, left ventricular; LVEF, left ventricular ejection fraction; NSVT, non-sustained ventricular tachycardia; PLN, phospholamban; RBM20, RNA-binding motif protein 20.
